# Design and Optimization of a Hybrid Railcar Structure with Multilayer Composite Panels

**DOI:** 10.3390/ma18215013

**Published:** 2025-11-03

**Authors:** Alessio Cascino, Enrico Meli, Andrea Rindi

**Affiliations:** Department of Industrial Engineering, University of Florence, 50139 Florence, Italy; enrico.meli@unifi.it (E.M.); andrea.rindi@unifi.it (A.R.)

**Keywords:** railway vehicle design, laminate optimization, finite element analysis, multilayer composite panels, lightweight design, structural optimization

## Abstract

Within contemporary railway engineering, manufacturers of rolling stock are increasingly focused on developing vehicles that combine reduced weight with enhanced reliability. This objective is largely motivated by the need to decrease energy demand and limit environmental impact, encouraging the integration of innovative materials and cut-ting-edge design strategies. The growing use of multilayer composite materials in the railway sector stems from their unique combination of high strength and low weight, making them particularly suitable for structural applications. This study investigates the structural performance and optimization of a hybrid car body system composed of an aluminum frame integrated with multilayer composite panels. A fully automated computational framework has been developed to generate and assess all possible stacking sequence permutations of the laminate plies, coupled with a high-fidelity finite element model of the car body. The methodology enables the evaluation of failure indices, including Maximum Stress, Tsai–Wu, and Interlaminar criteria, across a wide design space. A comprehensive assessment of both mechanical and dynamic performance has been carried out according to relevant railway standards, supporting the robustness and reliability of the proposed optimization framework. The results confirm the capability of the methodology to efficiently identify and compare multiple laminate configurations while maintaining compliance with structural and modal requirements. The optimized configurations demonstrated maximum Tsai–Wu values below 0.9, first-mode frequency variations below 0.5% and potential mass reductions of 25–45% on the selected components. This approach provides a powerful and versatile tool for the rapid optimization of lightweight hybrid structures in railway applications.

## 1. Introduction

The railway industry is undergoing a significant transformation, primarily fueled by the growing need for vehicles that are lighter, more energy-efficient, and environmentally sustainable. To meet these objectives, rolling stock manufacturers are increasingly turning to innovative structural solutions and the adoption of advanced materials capable of reducing mass while ensuring high safety and performance standards [1,2]. The adoption of advanced materials and structural optimization techniques has recently shown promising results in railway engineering, with several studies focusing on composite and sandwich panels for car body structures, evaluating and optimizing their mechanical performance. Using finite element software, mass reductions of around 30% have been obtained [3], while experimental campaigns on curved GFRP composites confirmed their impact resistance and overall suitability for railway use [4]. Additional optimization strategies, including topology, shape, and size refinements, have been applied to ribs and shells [5], and dynamic size optimization has been adopted in light rail vehicles to account for modal behavior [6], while further developments have even incorporated honeycomb-core panels in innovative car body structures [7]. Despite these advances, aluminum continues to be the standard reference material for railway car bodies due to its advantageous combination of high strength-to-weight ratio, excellent corrosion resistance, and good manufacturability [8]. However, the introduction of carbon fiber reinforced polymers (CFRPs) has opened new pathways for weight reduction while preserving structural performance, and current research is investigating their mechanical properties, long-term durability, and economic feasibility in comparison with aluminum [9,10,11,12,13]. Composite laminates are widely used in the automotive, aerospace, and marine industries due to their high strength-to-weight ratio, corrosion resistance, and its capability to be customized to meet specific performance demands. In the automotive sector, these materials are employed in structural and non-structural components such as body panels, headlight housings, under-the-hood parts, and interior elements, contributing to lighter, more fuel-efficient vehicles with improved crash performance and durability [14,15,16,17,18]. In aerospace, fiber metal laminates (FMLs) like glass laminate aluminum reinforced epoxy (GLARE) and aramid reinforced aluminum laminate (ARALL) are used in aircraft fuselages and structural panels, offering enhanced fatigue resistance and impact tolerance [19,20,21,22,23], while marine applications benefit from the durability and corrosion resistance of composite laminates, which are used in hulls, decks, and superstructures of ships and boats [24,25,26,27,28,29]. Despite their advantages, composite laminates face challenges such as complex failure modes (e.g., delamination, matrix cracking), high production costs, and the need for advanced damage detection and repair techniques, and ongoing research focuses on improving manufacturing efficiency, developing hybrid and natural fiber composites, and enhancing damage resistance and monitoring through intelligent computational methods [30,31,32,33]. Beyond the car body itself, the potential of lightweight design extends to other critical structural elements, such as bogie frames and bolster beams, where the combination of novel materials with optimization approaches has demonstrated significant improvements in these highly loaded components [34,35]. The railway sector’s shift toward lighter and more energy-efficient vehicles has therefore been accompanied by a growing reliance on advanced numerical models, and finite element modeling in particular is widely used to simulate operational conditions and evaluate structural behavior. One study designed and tested three different types of composite laminates to improve a railway component, providing a detailed mechanical characterization of the lamina [36], and when compared to an aluminum alloy solution, the resulting component achieved a 28% mass saving while maintaining acceptable tensile, fatigue, and impact resistance. Another research effort applied pultrusion technology to fabricate glass fiber reinforced polymer (GFRP) panels for medium-speed vehicles, successfully meeting EN12663-1:2015 requirements and obtaining a 35.5% reduction in weight relative to the initial steel-based design [37], while among the most recent investigations, a comparative study assessed aluminum and CFRP thin-walled structures for railway vehicle car bodies, analyzing static and dynamic responses through high-fidelity finite element models and optimization processes [38]. In summary, the literature analysis highlights that, although multilayer composite materials offer remarkable potential for railway applications thanks to their superior specific properties and high design flexibility, their use in industrial practice often lacks a systematic optimization of the stacking sequence. In this context, the present research introduces a new, rapid, and efficient methodology for the optimization of composite laminates intended for railway vehicles and potentially extendable to other sectors. The fully automated computational cycle, combined with an intuitive visualization of the results, enables designers to identify in just a few minutes the optimal stacking configuration that satisfies both regulatory requirements and structural performance targets, thus representing a significant advancement toward a more rational and performance-driven design of composite railway structures.

## 2. Methodology

Composite multilayer materials are increasingly gaining prominence in the design of modern railway vehicles due to their unique balance of low density, high strength, and excellent stiffness-to-weight ratio. Compared to traditional metallic solutions, composites allow significant weight reductions while maintaining adequate static and dynamic performance, thus contributing to energy efficiency, sustainability, and passenger safety. Their versatility lies in the possibility of tailoring the stacking sequence of plies to meet specific design requirements, offering a level of customization that metals cannot provide. These properties make multilayer composites a highly attractive alternative for the railway sector, where lightweight but structurally robust solutions are required to withstand demanding service loads and comply with rigorous safety standards. Despite these advantages, a recurring issue in current industrial practice is that multilayer laminates are often assembled following arbitrary criteria or simply based on supplier expertise, without a systematic approach to optimize the stacking sequence. This gap limits the ability of rolling stock manufacturers to fully exploit the potential of composites in car body design. In order to address this challenge, the methodology developed in this work provides a structured and automated framework to optimize the stacking sequence of composite laminates according to both the requirements of the EN 12663-1:2015 [39] and well-established performance indices available in the literature for multilayer materials. The strength of the proposed methodology lies in its high level of automation, combined with rigorous performance evaluation procedures applied at both the laminate and the car body structural level. The methodology is organized into five main phases:

(1) Finite element (FE) model of the car body structure. The process begins with the building of a high-fidelity FE model of the car body shell. This model accurately reproduces the geometry, boundary conditions, and loading scenarios, and serves as the reference framework for subsequent evaluations. Detailed modeling aspects are described in the following section.
(2) Definition of the composite stack. The second step involves defining the multilayer material, hereafter referred to as the stack. Each individual layer, denoted as ply, is characterized by its mechanical properties (elastic modulus, shear modulus, Poisson’s ratio, strength parameters, etc.) and introduced into the FE model through appropriate laminate modeling strategies.
(3) Numerical evaluation under European standard conditions. Once the stack is defined, the FE model is subjected to numerical simulations under the loading conditions prescribed by EN 12663-1:2015. Both static and dynamic performances are assessed for metallic and composite configurations. At this stage, the model provides insights into the global behavior of the structure, including modal properties such as natural frequencies and mode shapes.
(4) Optimization Process. The optimization phase is focused on identifying the most efficient stacking sequence for the composite laminate, according to the selected criteria. The procedure evaluates all possible ply arrangements, accounting for n! permutations, where n is the number of distinct plies. Redundancies are avoided by fixing the reference system of the stack, ensuring that no permutation is repeated. If identical plies are present, the number of evaluated configurations is automatically reduced, simplifying the process. The assessment of each configuration is based on Tsai–Wu, interlaminar, and Maximum Stress criteria, as well as on maximum failure index evaluations using percentile-based metrics.
(5) Global comparison of results. After completing the full cycle of n! iterations, a comprehensive comparison of all tested configurations is automatically generated. This final step identifies the optimal stacking sequence that maximizes structural efficiency while guaranteeing compliance with both European standards and design targets.

Through this methodology, an optimized laminate tailored to the specific car body structure can be systematically obtained. This approach is particularly valuable for complex and heavily loaded components such as railway car bodies, where the balance between weight reduction and structural integrity is crucial. However, the methodology could also be extended to a wide range of components that demand optimal performance under well-defined operating conditions, including those in the automotive, aerospace, and naval sectors.

## 3. Tram Platform Description

**Figure 1:**
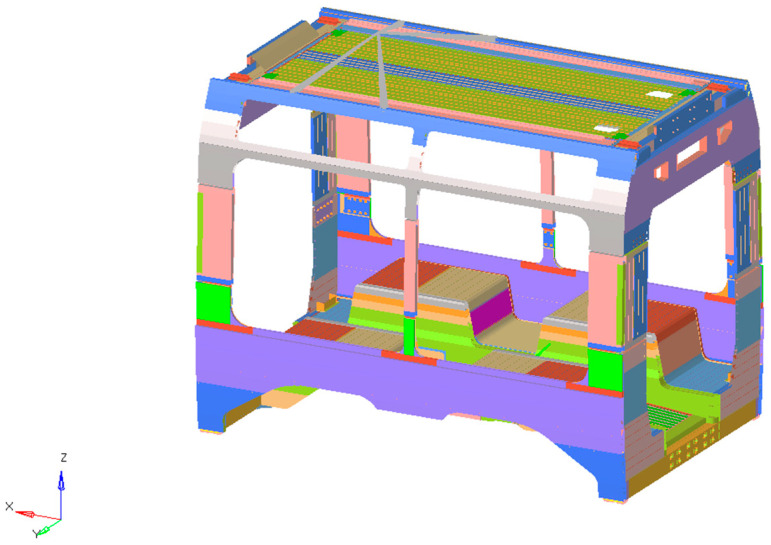
Car body structure.

The vehicle model examined in this study corresponds to a modern low-floor light rail vehicle, composed of five car bodies, or more, depending on the specific configuration. It is a vehicle specifically developed for urban service, which, as in the case of many con-temporary trams and some railway vehicles, introduces additional structural complexities arising from its design constraints. Lowering the floor level demands extensive changes to the underframe, often resulting in an intricate load conditions than that found in conventional layouts. To compensate for this, the structural arrangement is generally reinforced around highly stressed regions such as door openings and articulation joints. Moreover, the lack of a conventional high-floor structure necessitates alternative solutions for housing vital systems like traction units and braking equipment, which are frequently repositioned either on the roof or integrated within the bogies. These design measures ensure structural strength while optimizing passenger capacity and guaranteeing compliance with accessibility standards. The original vehicle construction employed aluminum alloy for the car bodies, while the driver’s cabins were manufactured using composite material and the bogie frames using construction steel. Concerning the car body structures, which are the primary focus of this study, two distinct configurations were identified. The first type interfaces directly with the bogies, is more compact in form, and its geometry allows for the integration of several underframe subsystems. The second type, which does not connect to the bogies, has a more elongated shape, providing the necessary space for passenger doors and vehicle access. All carbodies were essentially constructed using aluminum extruded profiles, mainly connected through welding. This approach was particularly employed for the roof and lower frame, whose geometries align with standard structural practices in railway vehicles. In contrast, the intermediate section, defining the window region and linking roof and underframe, was realized through riveting rather than welding. A detailed finite element model of this connection area was developed to verify its structural behavior. Figure 1 illustrates the first type of car body.

**Figure 2:**
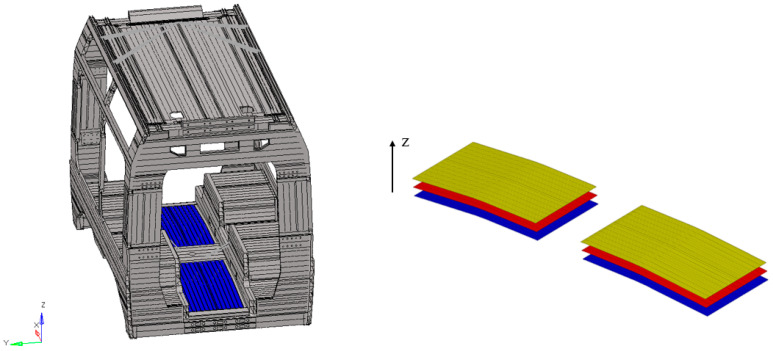
Localization of composite laminate within car body structure.

In order to improve performance both in terms of energy efficiency and lightweight design, evaluated as the ratio between weight and mechanical strength, this first car body type was selected for further investigation. As illustrated in Figure 2, multilayer laminates were incorporated in specific regions, resulting in a hybrid structural solution. In this context, several regions were identified as potential candidates for the application of composite laminates, including the side panels, the roof, and various internal structural areas such as the seating supports and the underframe. In the present research activity, attention was focused on the underframe, where the previous aluminum component was replaced with a composite laminate, allowing for an in-depth evaluation of its structural performance and weight reduction potential. This component is subjected to high bending loads, a condition in which composite laminates perform particularly well, thus offering significant opportunities for mass reduction.

### 3.1. Structural Materials in Comparison

A more detailed analysis of the aluminum alloy considered in this study, EN AW 6106 T6, is provided in accordance with the European standard EN 1999-1-1:2014 [40]. This material is part of the 6xxx series, distinguished by magnesium and silicon as its primary alloying elements. The main mechanical properties of EN AW 6106 T6 are reported in Table 1. This material offers an effective balance between strength, corrosion resistance, and weldability, making it particularly suitable for lightweight structural components. The T6 temper condition, achieved through solution heat treatment and artificial aging, further improves its yield and tensile strength, while maintaining good formability and fatigue resistance, key aspects for railway applications.

Composite laminates are increasingly adopted in the design of lightweight structural components for transportation systems due to their high specific strength, stiffness, and corrosion resistance. Their versatility lies in the ability to tailor the mechanical behavior of the final structure by combining multiple layers, or plies, each potentially characterized by different materials, fiber orientations, thicknesses, and geometries. A laminate typically consists of at least three plies, and the overall mechanical response depends on the stacking sequence, the material properties of each ply, and the interactions among them. This flexibility allows engineers to design structures that are optimized for specific load conditions, achieving a balance between weight reduction, mechanical performance, and manufacturing feasibility. In this research work, the objective was the development and optimization of a car body structure for tramway applications, focusing on the configuration of the composite laminate as the main subject of investigation. The study aims to demonstrate how the stacking sequence could be optimized to improve the structural behavior. Then, the proposed methodology considers a laminate composed of three plies made of three different materials, chosen to maximize the complexity of the optimization problem and to validate the effectiveness of the adopted approach. The mechanical properties and failure parameters values of each ply and the corresponding materials are summarized in Table 2 and Table 3.

### 3.2. FE Model Description and Simulation Settings

The finite element (FE) model of the car body provided a high-fidelity representation of the system, comprising approximately 1 million nodes. The car body structure, including all subassemblies, was modeled using a two-dimensional mesh of first-order QUAD4 shell elements. This meshing strategy was consistently applied to both the metallic and laminated composite structures. The main difference between the two lies in the properties assigned to the elements: for the laminate, a detailed definition of each ply’s characteristics was required. Additionally, to improve modeling quality and control over laminate parameters and optimization, a local dedicated subdomain was defined for the laminate, avoiding repetitions in permutational analyses. The average element size was approximately 16 mm, with local mesh refinements implemented to enhance stress evaluation quality and reliability. A detailed mesh sensitivity analysis was also performed, starting from an average element size of about 25 mm and progressively refining the mesh down to approximately 12 mm. The results showed a complete stabilization of the main stress and deformation parameters at the adopted 16 mm average element size, confirming the reliability and consistency of the selected discretization, in full agreement with the expected behavior. All rivets were modeled using a simplified approach, combining two rigid elements (RBE2 type, rigid body element type 2) with a suitable one dimensional beam element representative of the real connection element. Main equipment mounted on the vehicle was represented as localized masses connected to the structure via RBE3 elements. This configuration efficiently dis-tributes loads on the primary equipment connection points, reproducing interface conditions as realistically as possible. The interface between the laminate and the carbody, in the areas where a bonded joint would have been present, was modeled using a FREEZE contact applied only to the involved nodes. This approach allowed the correct transmission of all load conditions while maintaining a linear model, as the primary focus was placed on the methodology. The car body shell exhibited reduced thicknesses, accurately represented through the numerical formulation of the shell elements. Boundary conditions were applied in an isostatic arrangement, with vertical support provided by the secondary suspension, lateral constraints at the side pads, and longitudinal restraints at the rear cabin buffers. Numerical analysis were managed on a workstation equipped with an Intel(R) Xeon(R) CPU E5-2643 v4 @ 3.40 GHz and 32 GB of RAM. Based on the reference standard, the vehicle is classified in the P-V category, encompassing passenger vehicles, particularly light-rail vehicles. The standard mandates testing of the full vehicle model and specifies the loading conditions necessary for system verification. All the reference masses used in the numerical analysis were based on the UNI EN 15663:2019 standard [41]. This standard defines the terminology and classification of railway vehicle masses and provides guidance for their assessment. It ensures a uniform approach to mass categorization, enables comparison across different railway systems, and supports operation-al safety and efficiency. The standard identifies specific mass categories, including tare mass (C0), operational mass (C2), and maximum laden mass (C4). As noted previously, the static analysis, carried out according to the loading conditions specified in the Euro-pean standard, must always consider the entire vehicle, including all principal car body-to-car body interfaces and the associated relative movements. The selected reference load case represents the maximum vertical loading condition increased by 30% (including C4 mass), which constitutes the most severe scenario for the studied composite panel region. In this scenario, a density of six passengers per square meter, each with an approximate weight of 70 kg, was assumed, making it the critical reference condition for the structural evaluation and optimization of the laminate.

## 4. Results and Discussions

The present analysis focuses on a laminated composite panel designed for installation in the floor region of a railway vehicle. This component plays a critical structural and functional role, combining stiffness and impact resistance with stringent mass constraints. In railway design, where energy efficiency and dynamic performance are directly influenced by vehicle mass, a rapid and systematic evaluation of multiple laminate configurations becomes essential. The optimization framework adopted here enables automatic generation, analysis, and comparison of several stacking sequences, allowing the identification of the most efficient configuration according to selected criteria. In accordance with the reference standard, the investigation was carried out under the maximum vertical load condition, considered the most severe for the case studied in this research and generally recognized as the most demanding load scenario for most classes of railway vehicles.

### 4.1. Performance Indices and Assessment Criteria

In order to evaluate the structural integrity of the different laminate configurations under study, a unified failure metric, referred to as the Maximum Failure Index (FI_max), was defined. This index condenses the information from multiple classical composite failure criteria, as the Maximum Stress, Tsai–Wu, and Interlaminar Shear criteria, into a single scalar quantity representative of the most critical stress condition experienced within each finite element. The rationale behind this approach is that different regions of the laminate may fail according to different mechanisms (fiber breakage, matrix cracking, or delamination), and thus, the governing mode of failure at each point must be captured through the most severe among the active criteria. Formally, for each integration point, the three local indices were computed as follows. The Maximum Stress criterion assumes failure when any principal stress component exceeds its corresponding strength limit, and can be written as:

(1) $${FI}_{MaxStress}=max(\frac{\sigma_{1}}{X_{t}},-\;\frac{\sigma_{1}}{X_{c}},\;\frac{\sigma_{2}}{X_{t}},\;-\;\frac{\sigma_{2}}{X_{c}},\;\frac{\left|\tau_{12}\right|}{S})$$

where $\sigma_{1},\;\sigma_{2}$ are the normal stresses along the material directions, $\tau_{12}$ is the in-plane shear stress, and $X_{t},\;X_{c},\;Y_{t},\;Y_{c},\;S$ denote the corresponding tensile, compressive, and shear strengths. The Tsai–Wu criterion, which accounts for interaction effects between stress components, was implemented in the simplified polynomial form:

(2) $${FI}_{Tsai-Wu}=F_{1}\sigma_{1}+F_{2}\sigma_{2}+F_{11}{\sigma_{1}}^{2}+F_{22}{\sigma_{2}}^{2}+F_{66}{\tau_{12}}^{2}+2F_{12}\sigma_{1}\sigma_{2}$$

where $F_{1}=\frac{1}{X_{t}}-\frac{1}{X_{c}}$, $F_{2}=\frac{1}{Y_{t}}-\frac{1}{Y_{c}}$, $F_{11}=\frac{1}{X_{t}X_{c}}$, $F_{22}=\frac{1}{Y_{t}Y_{c}}$, $F_{66}=\frac{1}{S^{2}}$, and $F_{12}=-0.5\sqrt{F_{11}F_{22}}$. Finally, the Interlaminar criterion was evaluated across the interfaces between plies to capture potential delamination, expressed as:

(3) $${FI}_{Interlaminar}=max(\;\frac{\left|\tau_{1z}\right|}{\tau_{IL}},\;\frac{\left|\tau_{2z}\right|}{\tau_{IL}},)$$

where $\tau_{1z},\;\tau_{2z}$ are the interlaminar shear components and $\tau_{IL}$ is the interlaminar shear strength. The Maximum Failure Index was then defined as the envelope of all these competing failure mechanisms:

(4) $${FI}_{max}=max(\;{FI}_{MaxStress},\;{FI}_{Tsai-Wu},{FI}_{Interlaminar})$$

This formulation ensures that each element is characterized by a single non-dimensional indicator, where values $FI_{\max}<1$ correspond to safe conditions, and $FI_{\max}\geq 1$ indicate failure initiation according to at least one criterion. The resulting field of $FI_{\max}$ thus provides a comprehensive and conservative representation of the structural performance of the laminate, integrating both intralaminar and interlaminar failure modes into a unified visualization framework.

### 4.2. Mass Comparison and Optimization Framework

Table 2 summarizes the properties of the three candidate plies used in the stacking sequence permutations. The carbon/epoxy ply exhibits the highest stiffness and moderate density (1590 kg/m^3^), the glass/epoxy ply offers intermediate mechanical properties and a higher density (2010 kg/m^3^), whereas the aramid/epoxy ply is the lightest (1400 kg/m^3^) but with lower stiffness. Considering that each ply has a nominal thickness of 1 mm, the total thickness of each laminate configuration amounts to 3 mm, allowing a direct comparison with a 3-mm aluminum sheet (density ≈ 2700 kg/m^3^). For an equivalent surface area, the composite laminates yield a mass reduction between 25% and 45% compared to aluminum, depending on the sequence of the constituent plies. This significant weight advantage demonstrates the potential of hybrid laminates to achieve stiffness and strength performance comparable to metallic panels while substantially reducing structural mass, an essential factor in railway applications aimed at energy savings and dynamic efficiency. The optimization strategy automatically generates all n! permutations of the three-ply sequence (n = 3 → 6 unique stacking orders), each corresponding to a distinct configuration, denoted as stack_1 through stack_6. Each configuration is evaluated using finite element post-processing routines that compute the failure indices according to the selected criteria (Maximum Stress, Tsai–Wu, and Interlaminar Shear). The algorithm ensures that redundant configurations are avoided: if two or more plies are identical, equivalent stacking sequences are automatically eliminated from the analysis, thus improving computational efficiency. This systematic and automated approach allows each configuration to be compared on a common reference frame, highlighting the most efficient layup without manual intervention. The six analyzed stacks correspond to the following ply permutations (Table 4):

### 4.3. Tsai–Wu and Interlaminar Failure Index Distributions

The Tsai–Wu failure index plots provide an efficient and compact visualization of the distribution of failure index values across all finite elements in the structure for each laminate configuration. This representation makes it possible to instantly identify whether two or more configurations exhibit similar mechanical responses, when the scatter points overlap, or if one solution experiences localized peaks indicating critical stress concentrations. The Tsai–Wu criterion, being sensitive to multiaxial stress states, effectively captures complex interactions between longitudinal, transverse, and shear stresses within each ply.

**Figure 3:**
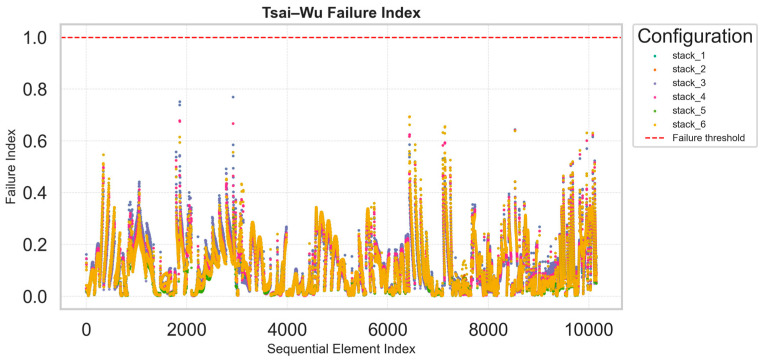
Distribution of the Tsai–Wu failure index across all finite elements.

From the comparative plots, the overall distribution of Tsai–Wu indices remains below unity for most configurations (Figure 3), confirming that all stacks are within the allowable strength limits. Some local peaks are observed: stack_3 shows a maximum value of approximately 0.85, while stack_5 reaches values close to 0.9, suggesting the presence of localized stress intensification. These peaks correspond to specific regions of geometric discontinuity in the panel.

**Figure 4:**
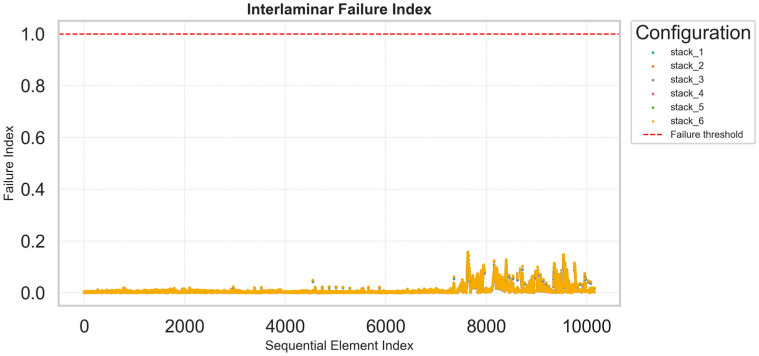
Distribution of the Interlaminar failure index across all finite elements.

The interlaminar failure index plots exhibit a more uniform and smoother distribution, with maximum values generally lower than 0.2 across all configurations (Figure 4). This indicates limited risk of delamination and satisfactory shear transfer between plies, consistent with the relatively balanced nature of the investigated layups. The ability to compare these indices across multiple stacking sequences in a single plot is a major advantage of the adopted methodology: instead of opening and post-processing n different FEM models individually, engineers can identify the most critical regions and configurations directly from these synthetic plots. Each graph is associated with an underlying dataset that reports, for every element of the structure, the computed failure indices and corresponding spatial coordinates. This allows subsequent detailed investigation within the finite element model once the optimal configuration has been identified. The key benefit of this procedure lies in rapid and consistent comparison: the optimization process aims not at the highest local accuracy for each model but at an efficient and reliable screening of multiple design options. Once the most promising stack is selected, it can be reimported into the FEM environment for refined, localized verification.

### 4.4. Percentile and Statistical Evaluation of Maximum Failure Index

**Figure 5:**
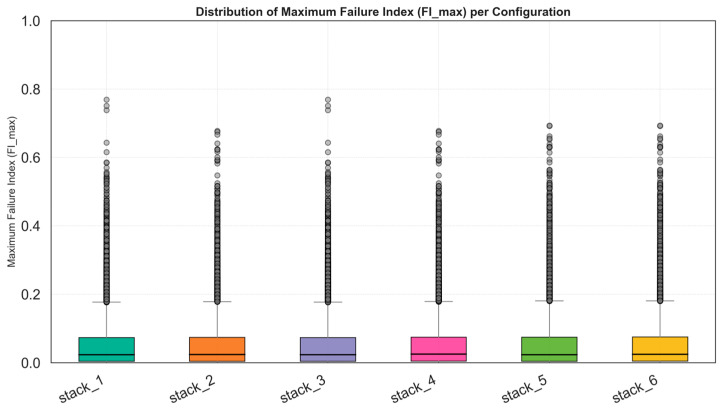
Distribution of Maximum failure index (FI_max) for all stacking permutations.

The percentile plots (p90 and p95) and the boxplot representations provide a complementary statistical perspective of the numerical results. These visualizations display not only the peak or mean values of the failure indices but the entire distribution across the structure, allowing an assessment of variability and robustness. In the boxplots, each rectangle represents the interquartile range (IQR), the interval between the 25th and 75th percentiles, with the central horizontal line marking the median. The whiskers indicate the overall spread of the data (typically within 1.5 × IQR), while individual dots outside this range denote localized stress peaks or outlier elements within the laminate. A narrow box with a low median suggests a uniform and well-distributed stress state, desirable for fatigue durability and structural efficiency. The percentile comparison graph (p90–p95) quantifies the tail of the distribution, i.e., the highest 10% and 5% of the elements by failure index value. This allows direct comparison of the most critical regions across configurations. As shown in Figure 5, the analyzed cases, stack_2, stack_4, stack_5 and stack_6 consistently exhibit the lowest p95 values, confirming their superior performance and uniformity. Conversely, stack_1 and stack_3 show higher percentile values, indicating less favorable stress distribution and greater sensitivity to local effects.

These statistical indicators, combined with the Tsai–Wu and interlaminar plots, provide a comprehensive evaluation of laminate performance. They allow the designer to balance strength considerations in a single, integrated optimization loop, reducing design iteration time and improving decision reliability.

### 4.5. Stress Distribution and Modal Behavior Assessment

**Figure 6:**
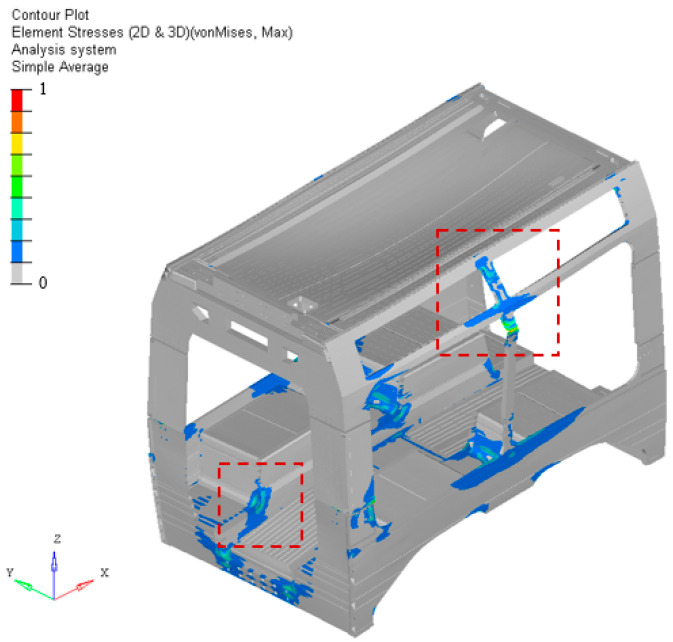
Stress distribution and main stress localization on the car body shell.

**Figure 7:**
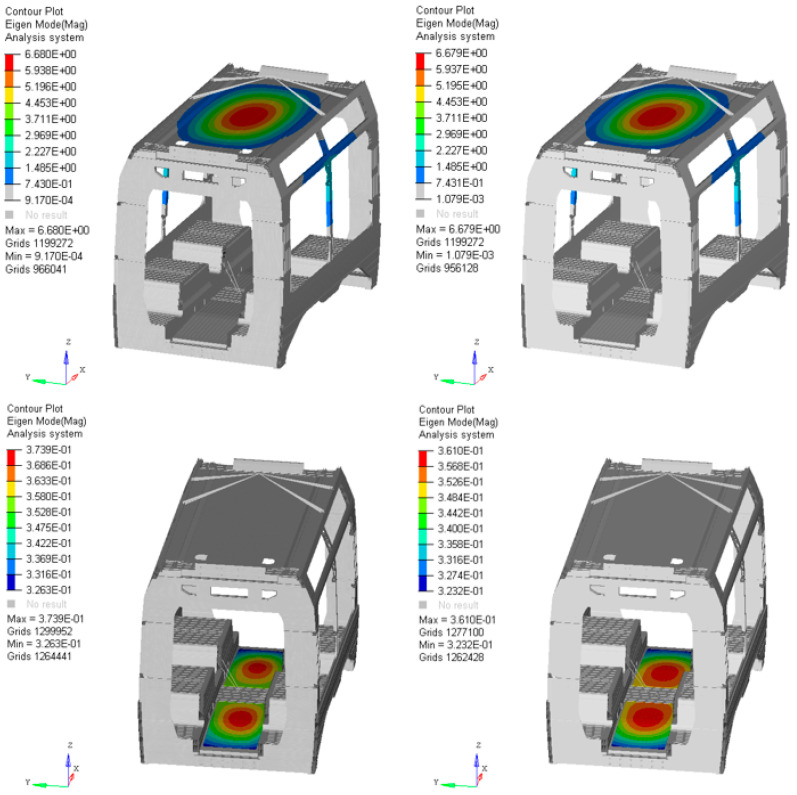
Comparison of first mode shape. Global view and local view of underframe. Original structure on the left, redesign structure on the right.

The modal and stress analyses reveal that variations in the stacking sequence have a negligible influence on the global structural response of the system. Across all investigated configurations, both the maximum and average stress levels remain nearly unchanged, with deviations well within the expected numerical tolerance. Similarly, the natural frequencies obtained from the modal analysis exhibit only minimal variations among the different stacking sequences, confirming that the structural stiffness and dynamic behavior of the overall system are not significantly affected by local modifications within the laminate. This outcome demonstrates that, while local ply orientation and stacking order influence the distribution of failure indices at the microscopic level, their effect on the macroscopic modal characteristics and global stress field of the car body floor structure is marginal. The structural boundary conditions and global stiffness of the car body dominate the response, making the laminate optimization primarily a local refinement process aimed at minimizing failure risk without altering the global dynamic integrity of the system. For the two main stress concentrations highlighted in Figure 6, the calculated material utilization factors are lower than one, thus acceptable with respect to the allowable limits and safety factors prescribed by the reference standard. The normalized stress distribution shows two main concentration zones, one located in the floor area and the other on the vertical side wall. In addition, the dynamic analysis reported in Figure 7 confirms that the design variations have negligible impact on the overall modal behavior of the car body. The global first vibration mode remains unchanged, and the local displacement distribution on the underframe is practically equivalent, including the maximum values. The first eigenvalue, around 30 Hz, exhibited a difference of less than 0.5%, further supporting the conclusion that the optimized stacking configuration maintains both structural and dynamic performance within acceptable limits. The overall mechanical behavior is consistent with expectations and confirms the structural suitability of the design.

## 5. Conclusions

The research presented in this work focuses on the optimization of a hybrid car body structure for a tramway application, in which a multilayer composite laminate has been integrated within an aluminum frame. This solution represents a practical implementation of lightweight design principles, aiming to achieve a significant mass reduction while preserving the mechanical strength, stiffness, and fatigue performance required by railway standards. To this end, a fully automated optimization methodology was developed to explore all possible stacking sequence permutations of the laminate plies and evaluate their mechanical performance. The process is directly coupled with a high-fidelity finite element model of the car body, enabling the extraction of stress, failure indices, and modal characteristics for each configuration without manual intervention. This framework allows a complete evaluation of n! stacking alternatives, effectively avoiding redundant configurations and significantly accelerating the design process. The analysis of the results demonstrates that the variation in stacking sequence induces only marginal differences in both the stress field and the natural frequencies of the structure. The maximum value of the Tsai–Wu failure index across all configurations was 0.9, while the variation observed in the first vibration frequency was lower than 0.5%. Utilization coefficients remained below 1. The mechanical and dynamic behavior of the hybrid car body therefore remain consistent across all configurations, confirming the robustness of the design and its compliance with operational requirements. Moreover, the selected composite laminate allows a potential mass reduction of 25–45% on the chosen components, depending on the stacking sequence. The visualization tools introduced, including the Tsai–Wu and interlaminar failure index plots and the maximum failure index percentile-based evaluations, have proven to be particularly effective in supporting rapid comparison among multiple solutions, providing engineers with a comprehensive yet intuitive overview of the laminate performance. Overall, the methodology constitutes an efficient and reliable approach for the structural optimization of hybrid aluminum–composite systems, reducing design time and ensuring that all configurations are assessed under consistent boundary conditions. Future developments of this research will focus on extending the methodology to fiber orientation optimization, where the in-plane direction of reinforcement is varied to maximize stiffness and minimize failure risk for specific load cases. Further studies will also explore the effect of variable ply thickness on the laminate response, enabling more advanced multi-objective optimization schemes. Finally, a sensitivity analysis on the key material and geometric parameters will be performed to quantify their combined influence on the global performance, thereby enhancing the robustness and predictive capability of the proposed design framework.

## Figures and Tables

**Table 1:** Mechanical properties of the aluminum alloys [40].

Material	Density	Young’s Modulus	Proof Strength (0.2%)	Ultimate Tensile Strength	Proof Strength (0.2%)	Ultimate Tensile Strength	Poisson’s Ratio
			Base Material		Weld Material		
	[kg/m^3^]	[N/mm^2^]	[N/mm^2^]		[N/mm^2^]		[-]
EN AW 6106 T6	2700	70,000	200	250	95	160	0.30

**Table 2:** Mechanical properties of plies.

Ply	Material	Thickness [mm]	Density [kg/m^3^]	E_1_ [GPa]	E_2_ [GPa]	ν_12_ [GPa]	G_12_ [GPa]	G_13_ [GPa]	G_23_ [GPa]
1	Carbon fiber/Epoxy	1	1590	135	8.0	0.30	5.0	5.0	3.0
2	Glass fiber/Epoxy	1	2010	40	10.0	0.25	4.5	4.5	3.5
3	Aramid fiber/Epoxy	1	1400	25	7.5	0.35	3.0	3.0	2.0

**Table 3:** Failure parameters values for the plies.

Ply	Material	Xt [GPa]	Xc [GPa]	Yt [GPa]	Yc [GPa]	S [GPa]	τ_IL_ [GPa]
1	Carbon fiber/Epoxy	1500	800	60	160	80	60
2	Glass fiber/Epoxy	400	250	80	150	50	40
3	Aramid fiber/Epoxy	1200	700	40	140	30	35

**Table 4:** Stack permutations.

Stack	Sequence (Bottom → Top)
stack_1	Carbon/Glass/Aramid
stack_2	Carbon/Aramid/Glass
stack_3	Glass/Carbon/Aramid
stack_4	Glass/Aramid/Carbon
stack_5	Aramid/Carbon/Glass
stack_6	Aramid/Glass/Carbon

## Data Availability

The original contributions presented in this study are included in the article. Further inquiries can be directed to the corresponding author.
